# Should Physical Activity Recommendations Be Ethnicity-Specific? Evidence from a Cross-Sectional Study of South Asian and European Men

**DOI:** 10.1371/journal.pone.0082568

**Published:** 2013-12-11

**Authors:** Carlos A. Celis-Morales, Nazim Ghouri, Mark E. S. Bailey, Naveed Sattar, Jason M. R. Gill

**Affiliations:** 1 Institute of Cardiovascular and Medical Sciences, College of Medical, Veterinary and Life Sciences, University of Glasgow, Glasgow, United Kingdom; 2 Human Nutrition Research Centre, Institute for Ageing and Health, Newcastle University, Newcastle on Tyne, United Kingdom; 3 School of Life Sciences, College of Medical, Veterinary and Life Sciences, University of Glasgow, Glasgow, United Kingdom; University of Sao Paulo, Brazil

## Abstract

**Background:**

Expert bodies and health organisations recommend that adults undertake at least 150 min.week^−1^ of moderate-intensity physical activity (MPA). However, the underpinning data largely emanate from studies of populations of European descent. It is unclear whether this level of activity is appropriate for other ethnic groups, particularly South Asians, who have increased cardio-metabolic disease risk compared to Europeans. The aim of this study was to explore the level of MPA required in South Asians to confer a similar cardio-metabolic risk profile to that observed in Europeans undertaking the currently recommended MPA level of 150 min.week^−1^.

**Methods:**

Seventy-five South Asian and 83 European men, aged 40–70, without cardiovascular disease or diabetes had fasted blood taken, blood pressure measured, physical activity assessed objectively (using accelerometry), and anthropometric measures made. Factor analysis was used to summarise measured risk biomarkers into underlying latent ‘factors’ for glycaemia, insulin resistance, lipid metabolism, blood pressure, and overall cardio-metabolic risk. Age-adjusted regression models were used to determine the equivalent level of MPA (in bouts of ≥10 minutes) in South Asians needed to elicit the same value in each factor as Europeans undertaking 150 min.week^−1^ MPA.

**Findings:**

For all factors, except blood pressure, equivalent MPA values in South Asians were significantly higher than 150 min.week^−1^; the equivalent MPA value for the overall cardio-metabolic risk factor was 266 (95% CI 185-347) min.week^−1^.

**Conclusions:**

South Asian men may need to undertake greater levels of MPA than Europeans to exhibit a similar cardio-metabolic risk profile, suggesting that a conceptual case can be made for ethnicity-specific physical activity guidance. Further study is needed to extend these findings to women and to replicate them prospectively in a larger cohort.

## Introduction

Two of the major modifiable risk factors for type 2 diabetes and cardiovascular disease are obesity [1], [2] and a low level of physical activity [3], [4]. Because of this, national and international bodies have made recommendations regarding appropriate levels for body mass index (BMI) [5], waist circumference [5], and physical activity [6]–[8], to minimise risk of vascular and metabolic disease.

Over recent years it has become apparent that the BMI and waist circumference cut-points used to define obesity in populations of white European origin are not appropriate for a number of non-white populations. In particular, for South Asians – who form 20% of the world’s population – substantially lower BMIs and waist circumferences are needed to confer equivalent cardio-metabolic risk factor profiles to those observed in populations of white European origin [9], [10]. Data from the Canadian SHARE and UK ADDITION-Leicester cohorts indicate that migrant South Asians require BMI values of ∼21–23 kg.m^−2^ to have equivalent glycaemia, and ∼22–26 kg.m^−2^ to have equivalent lipid profiles, to those observed in white Europeans at the conventional obesity threshold of 30 kg.m^−2^ [9], [10]. Findings such as these have led to recent consensus statements from academic groups in two countries recommending that the threshold for obesity should be lowered to BMI 25 kg.m^−2^ in South Asian populations [11], [12]. Similar findings have been reported for waist circumference [12]–[14]. Furthermore, recent guidance from the UK National Institute for Health and Care Excellence (NICE) has recommended using lower thresholds for BMI to trigger action to prevent type 2 diabetes amongst Asian populations (23 kg.m^−2^ (*vs* 25 kg.m^−2^ for white populations) for ‘increased risk’ and 27.5 kg.m^−2^ (*vs* 30 kg.m^−2^) for ‘high risk’) [15].

While it is now generally accepted that recommended thresholds for BMI and waist circumference should differ according to ethnicity, current recommendations for physical activity are not ethnicity-specific [6]–[8], and largely emanate from work done in populations of white European descent [16]. However, preliminary data suggest that the dose-response relationship between physical activity and risk factors for metabolic disease differs substantially between populations of different ethnicities [17]. South Asians are a group at increased risk of diabetes and cardiovascular disease [18], who have lower levels of cardiorespiratory fitness [19]–[22] and a reduced ability to oxidise fat during exercise [19], compared to white Europeans with equivalent levels of physical activity. Thus, the appropriate level of physical activity for health in South Asians may differ from the current recommendation of at least 150 minutes of moderate intensity (or 75 minutes of vigorous intensity) physical activity per week, with this activity performed in bouts of at least 10 minutes duration [6]–[8]. This has important implications for physical activity guidance provided to the South Asian public and to healthcare professionals advising South Asian patients. Thus, main aim of the work reported here was to determine the level of physical activity required in UK South Asians to elicit an equivalent cardio-metabolic risk profile to that observed in Europeans achieving the current guideline level of physical activity.

## Methods

### Participants

Data collected from all South Asian (defined as having both parents of Indian, Pakistani, Bangladeshi or Sri Lankan origin) and European (both parents of white European origin) men recruited to the cross-sectional **C**arotid **U**ltrasound and **R**isk of **V**ascular disease in **E**uropeans and **S**outh Asians (CURVES) study, who had valid data for objectively monitored physical activity, were included in this analysis (n = 75 South Asians and n = 83 Europeans). All participants were aged 40–70, living in the UK, and did not have coronary heart disease, cerebrovascular disease, peripheral vascular disease, or diabetes. They were recruited by local advertising/word of mouth and by writing to potentially eligible participants identified from four local primary care practice databases. The study was approved by the West of Scotland Research Ethics Committee and conducted according to the principles expressed in the Declaration of Helsinki. All participants gave written informed consent. Full descriptive details of this cohort have been described previously [22].

### Anthropometry

Height, body mass, and waist circumference (midpoint between the lower costal margin and iliac crest) were measured in all participants by the same person, trained in undertaking anthropometric measurements in accord with international standard protocols [23].

### Measurement of physical activity

Participants wore accelerometers (Actigraph G3TX+ or ActiTrainer; ActiGraph LLC, Pensacola, FL, USA) on the left or right hip at all times, except when showering, swimming and sleeping, for seven consecutive days to objectively assess physical activity levels. Vertical axis accelerometer counts were summarized in 60-second epochs and Freedson cut-points used to define intensity domains [24]. Data from participants with at least 10 hours of daily accelerometer wear time for 4 days were included in the analysis. Non-wear was defined by intervals of at least 60 minutes of zero activity counts [25]. Wear time was calculated by subtracting non-wear time from 24 hours. Current physical activity guidelines state that physical activity should be performed in bouts of at least 10 minutes duration [6]–[8]. We therefore calculated amount of physical activity of at least moderate intensity undertaken in bouts of at least 10 minutes (MPA_bouts_), with an allowance within a bout for interruptions of up to 2 minutes below the moderate intensity threshold [25], and used this physical activity variable for our data analysis.

### Blood biochemistry and blood pressure measurements

Venous blood samples were collected after an overnight fast of 10–12 hours. Glucose, HbA1c, total cholesterol, HDL cholesterol and triglyceride (TG) were analysed as routine samples on the day of collection in the National Health Service Biochemistry Laboratory in Glasgow, using standard automated enzymatic (for glucose and lipids) and HPLC (HbA1c) methods. This laboratory participates in the U.K. National External Quality Assessment Service (UKNEQAS) scheme and all tests had inter-assay CVs <5%. Plasma was stored at -80°C for the subsequent measurement of insulin, using commercially available enzyme-linked immunoassays (ELISA) (Mercodia AB, Uppsala, Sweden), in a single batch at the end of the study.

Blood pressure was measured on the left arm after at least 15 minutes of seated rest using an automated blood pressure monitor (Omron HEM705 CP, Omron Healthcare UK Limited, Milton Keynes, UK) which has been validated according to the European Society of Hypertension International Protocol [26]. The mean of four blood pressure readings was used in analysis.

These eight variables (i.e. glucose, HbA1c, total cholesterol, HDL cholesterol, TG, insulin and systolic and diastolic blood pressure) were selected as biomarkers for the characterisation of cardio-metabolic risk in our analyses as they have all been shown to be predictive of future cardiovascular disease and/or diabetes in large cohort studies [27], [28].

### Data analysis

**Figure 1:**
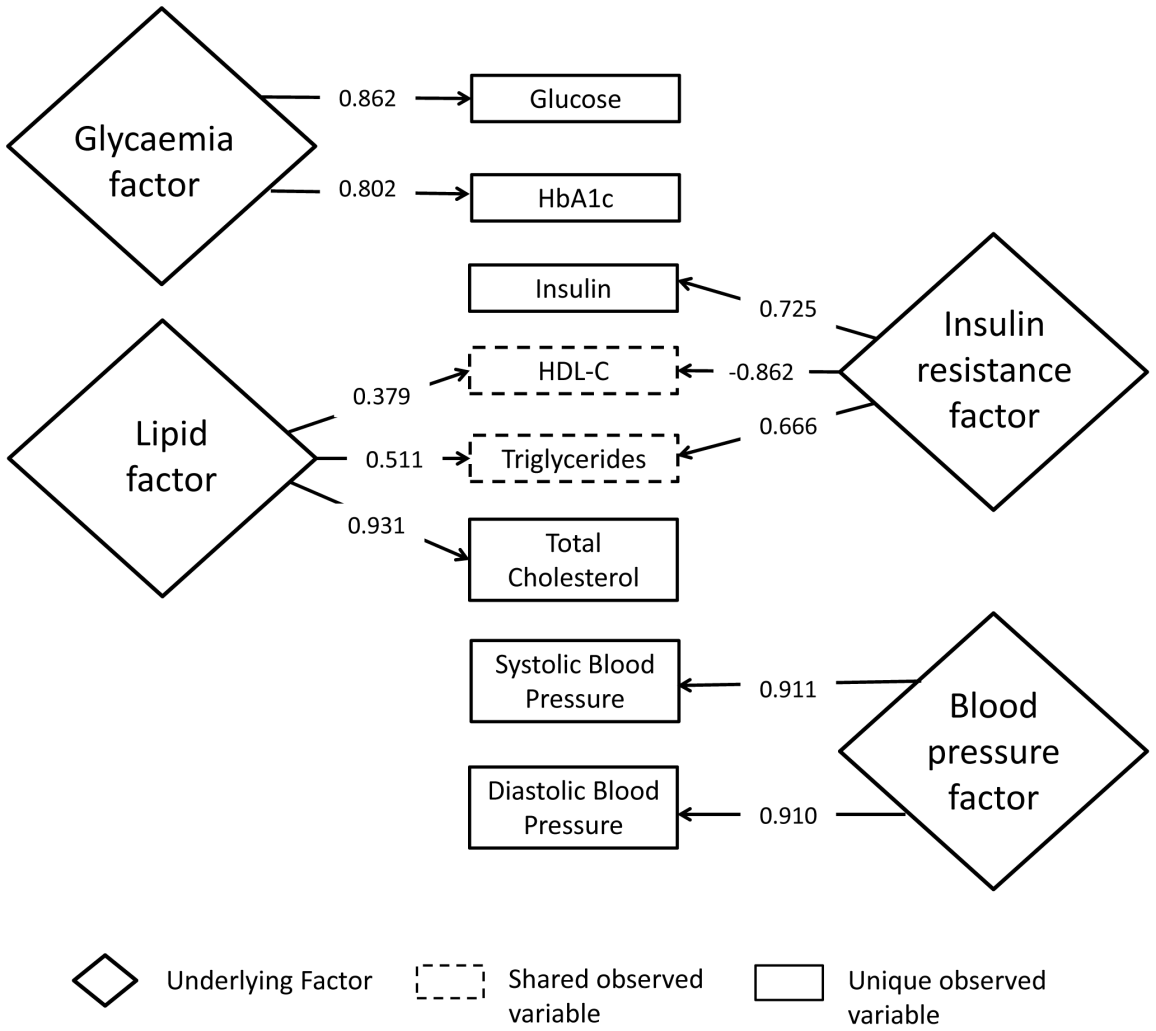
Relationship between measured variables and underlying latent factors. Numbers in arrows show factor loadings. Relationships between latent factors and measured variables only shown for rotated factor loadings >0.32 (i.e. where observed variable explains >10% of the variance in the latent factor (0.32^2^ = 0.10)). See methods for further details.

Statistical analysis was performed using Stata (Version 11.0, StataCorp LP, College Station). Using an approach described previously [9], [10], factor analysis was used to reduce the range of measured cardio-metabolic risk biomarkers into underlying latent factors. Biomarkers with rotated loadings >0.32 for principal components (i.e. explaining >10% of the variance in a factor (0.32^2^ = 0.10)) in these analyses were clustered into summary factors. Four separate factors emerged summarising glycaemia (glucose, HbAlc), insulin resistance (insulin, HDL cholesterol, triglycerides), lipid metabolism (total cholesterol, HDL cholesterol, triglycerides), and blood pressure (systolic and diastolic blood pressure). Figure 1 provides a visual representation of the relationships between the measured biomarkers and the underlying factors. We also derived an overall cardio-metabolic risk factor which comprised a summary measure of all eight measured risk factors. Regression models, adjusted for age, BMI, daily accelerometer wear time, and number of days of accelerometer wear, were fitted with each summary factor as the dependent variable; and ethnicity, time spent in MPA_bouts_, and the interaction term between ethnicity and MPA_bouts_, as the independent variables. Predicted values for each factor in European men for an MPA_bouts_ level of 150 min.week^−1^ were then calculated. We then determined the MPA_bouts_ level which gave the same predicted values in each factor for South Asian men, to provide equivalent threshold values. 95% confidence intervals (95% CI) for the equivalent South Asian thresholds were calculated from the 95% confidence bands from the regression lines. To provide external validation of our dataset/analysis by enabling comparison with previously published work on ethnicity specific obesity cut-points, we also determined (age-adjusted) equivalent South Asian cut-points for a BMI of 30 kg.m^−2^ and for a waist circumference of 102 cm in European men, using the same approach. All models were checked to ensure normality of the residuals and the linearity of the relationship between predictors and outcome. As there may be ethnic differences in HbA1c levels that are independent of glycaemia [29], [30], we performed a sensitivity analysis, removing HbA1c from the glycaemia and overall cardio-metabolic risk factors. Values are presented as means with 95% CI unless otherwise stated.

## Results

Detailed descriptive data for the South Asian and European men in this sample have been previously reported [22]. Descriptive data directly relevant to the present analyses are shown in Table 1. There were no significant differences between the groups in age, BMI or waist circumference. The South Asian group undertook less physical activity than Europeans, and had higher glucose, HbA1c and insulin concentrations, lower total and HDL cholesterol concentrations, and higher diastolic blood pressure.

**Table 1:** Descriptive data for the sample cohort.

	South Asians	European	p-value
	(n = 75)	(n = 83)	
Age (years)	48.5±7.1	49.9±6.7	0.19
Body mass (kg)	82.0±11.7	86.2±14.4	0.04
Height (m)	1.74±0.07	1.78±0.06	<0.0001
BMI (kg.m^−2^)	27.1±3.7	27.1±4.5	0.97
Waist circumference (cm)*	96.7±10.4	96.3±11.7	0.77
Moderate-to-vigorous physical activity (min.week^−1^)*	181.3±116.0	298.5±174.5	<0.0001
MPA_bouts_ (min.week^−1^)*	54.4±78.2	128.2±115.2	<0.0001
Accelerometer wear time (hours.day^−1^)	13.7±1.4	14.4±1.2	0.003
Number of valid days of accelerometer wear (days)	5.7±1.1	6.2±0.9	0.003
Glucose (mmol.l^−1^)*	5.3±0.6	5.1±0.3	0.016
HbAlc (mmol.mol^−1^)*	38.5±4.7	35.5±3.0	<0.0001
HbA1c (%)*	5.7±0.4	5.4±0.3	<0.0001
Insulin (mmol.l^−1^)*	13.8±6.6	9.4±5.2	<0.0001
Total cholesterol (mmol.l^−1^)	5.3±0.9	5.6±1.0	0.04
HDL cholesterol (mmol.l^−1^)*	1.2±0.2	1.4±0.3	0.0002
Triglycerides (mmol.l^−1^)*	1.6±0.9	1.5±0.9	0.27
Systolic blood pressure (mm Hg)*	128±16	128±13	0.64
Diastolic blood pressure (mm Hg)*	78±9	75±7	0.02

Values are mean ­­­­­­­± SD. MPA_bouts_: ≥ moderate-intensity exercise performed in ≥ 10-minute bouts. *statistical analysis performed on log transformed data.

### Equivalent physical activity levels

**Figure 2:**
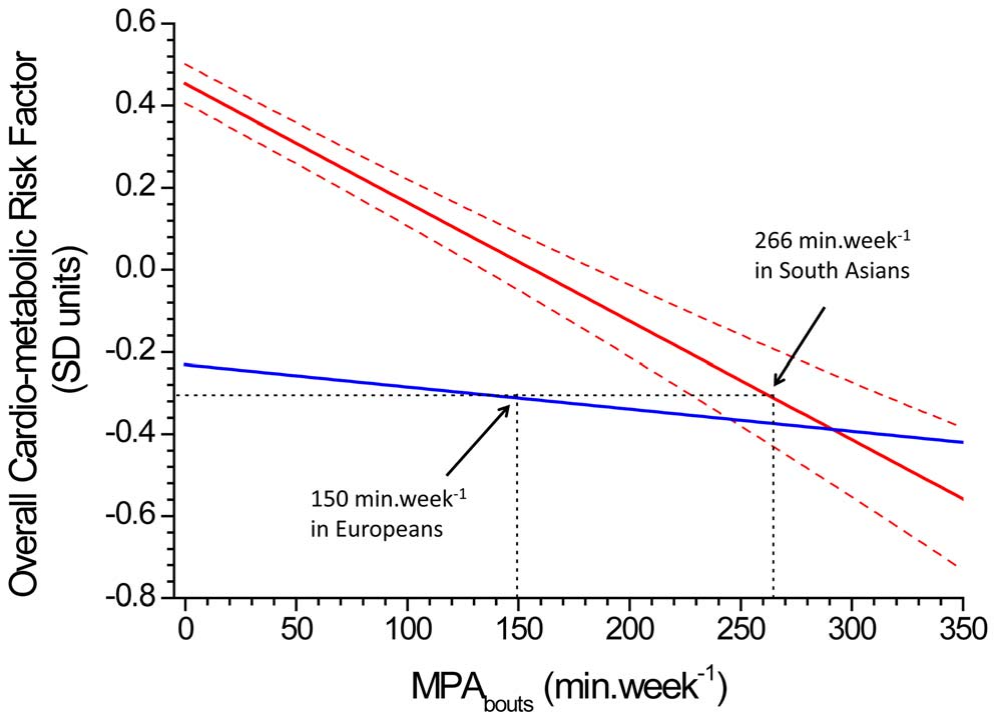
Relationship between the overall cardio-metabolic risk factor and level of physical activity of at least moderate intensity, performed in bouts of at least 10 minutes (MPA_bouts_) in South Asians (solid red line) and Europeans (solid blue line). 266 min.week^−1^ of MPA_bouts_ in South Asians gave an equivalent cardio-metabolic risk factor level to that observed in Europeans undertaking 150 min.week^−1^ of MPA_bouts_. Dotted red lines represent the 95% confidence bands around the regression line for South Asians. These bands were used to calculate the 95% CI around the equivalent level of MPA_bouts_ in South Asians. Regression lines are adjusted for age, BMI, daily accelerometer wear time, and number of days of accelerometer wear.

Table 2 shows the percentage variance in each factor explained by ethnicity and by MPA_bouts_, and MPA_bouts_ values for physical activity in South Asians which are equivalent to 150 min.week^−1^ of MPA_bouts_ in Europeans. Figure 2 displays how these threshold values were calculated, using the overall cardio-metabolic risk factor as an illustration. Ethnicity explained a substantial proportion of the variance (up to 10%) in all of the latent factors (except blood pressure factor, where it explained only 1.1% of the variance). The contribution of MPA_bouts_ to the variance in all of the latent factors was of similar magnitude to the ethnicity effect, with 9.5% of the variance in the overall cardio-metabolic risk factor explained by MPA_bouts_. Equivalent threshold values for MPA_bouts_ were significantly higher than 150 min.week^−1^ in South Asians, for the glycaemia factor (299 (221 to 394) min.week^−1^), insulin resistance factor (283 (239 to 342) min.week^−1^), lipid factor (364 (276 to 576) min.week^−1^), and overall cardio-metabolic risk factor (266 (228 to 313) min.week^−1^), but did not differ significantly from 150 min.week^−1^ for the blood pressure factor (118 (95 to 156) min.week^−1^). Removing HbA1c reduced the equivalent MPA_bouts_ value in South Asians for the glycaemia factor to 211 (163 to 335) min.week^−1^ and reduced the equivalent MPA_bouts_ value for the overall cardio-metabolic risk factor to 197 (160 to 224) min.week^−1^.

**Table 2:** Variance explained by physical activity of at least moderate intensity, performed in bouts of at least 10 minutes (MPA_bouts_), and derived value for MPA_bouts_ in South Asian men equivalent to 150 min.week^−1^ of MPA_bouts_ in European men, for glucose, insulin resistance, lipid, blood pressure and overall cardio-metabolic risk factors.

			Equivalent MPA_bouts_ value (min.week^−1^)	
	Variance in factor explained by ethnicity (%)	Variance in factor explained by MPA_bouts_ (%)	European (n = 83)	South Asian (n = 75)
Glycaemia factor	10.0	3.8	150	299 (211 to 394)
Glycaemia factor (excluding HbAlc)	3.7	2.3	150	211 (163 to 335)
Insulin resistance factor	9.9	9.1	150	283 (239 to 342)
Lipid factor	6.1	4.8	150	364 (276 to 578)
Blood pressure factor	1.1	2.3	150	118 (95 to 156)
Overall cardio-metabolic risk factor	9.1	9.5	150	266 (228 to 313)
Overall cardio-metabolic risk factor (excluding HbAlc)	5.7	8.9	150	197 (160 to 224)

Values are mean (95% CI). Factors represent single summary variables derived from factor analysis. The glycaemia factor includes fasting glucose and HbA1c; the insulin resistance factor includes insulin, HDL cholesterol, triglycerides; the lipid factor includes total cholesterol, HDL cholesterol, triglycerides; the blood pressure factor includes systolic and diastolic blood pressure; and the overall cardio-metabolic risk factor includes glucose, HbA1c, insulin, total cholesterol, HDL cholesterol, triglycerides and systolic and diastolic blood pressure. Models were adjusted for age, BMI, daily accelerometer wear time, and number of days of accelerometer wear.

### BMI and waist circumference cut-points

Cut-point values equivalent to a BMI of 30 kg.m^−2^ in Europeans, were 24.3 (22.1 to 26.9) kg.m^−2^ for the glycaemia factor, 23.3 (20.5 to 26.4) kg.m^−2^ for the insulin resistance factor, 23.3 (20.0 to 27.1) kg.m^−2^ for the lipid factor, 28.3 (24.1 to 30.5) kg.m^−2^ for the blood pressure factor, and 23.1 (20.9 to 27.6) kg.m^−2^ for the overall cardio-metabolic risk factor, when all participants were included in the analysis. Removing HbA1c from the glycaemia and overall cardio-metabolic risk factors, in sensitivity analysis, reduced the cut-point values for South Asians for the glycaemia factor to 22.1 (19.7 to 26.8) kg.m^−2^ and for the overall cardio-metabolic risk factor to 23.0 (20.3 to 27.2) kg.m^−2^.

For a waist circumference of 102 cm in European men, equivalent cut-point values in South Asians were 90.4 (85.1 to 97.3) cm for the glycaemia factor, 87.0 (81.6 to 91.4) cm for the insulin resistance factor, 85.3 (79.5 to 92.1) cm for the lipid factor, 100.7 (93.0 to 105.6) cm for the blood pressure factor, and 88.2 (81.1 to 92.7) cm for the overall cardio-metabolic risk factor. Removing HbA1c increased the glycaemia cut-point value to 94.9 (86.9 to 100.5) cm, and the overall cardio-metabolic risk factor cut-point to 88.6 (80.8 to 94.9) cm.

## Discussion

This study demonstrates, for the first time, that, for a given age and BMI, South Asian men may need to undertake greater levels of physical activity to exhibit a similar cardio-metabolic risk profile to that observed in European men undertaking the level of physical activity (150 min.week^−1^ of MPA_bouts_) currently recommended in physical activity for health guidance [6]–[8]. This suggests that there is a clear conceptual case for physical activity guidelines to be ethnicity-specific, in an analogous manner to that widely accepted for obesity cut-points [12]–[14]. Lending external validity to our work, our analyses also confirm previously reported findings that lower BMI and waist circumference cut-points are required in South Asian men to confer similar cardio-metabolic risk profiles to European men at conventional obesity thresholds (i.e. BMI 30 kg.m^−1^ and waist circumference 102 cm) [9], [10].

Low levels of physical activity are a major public health concern. It is estimated that 9% of deaths worldwide – more than can be attributed to smoking – are due to physical inactivity [31]. The 150 min.week^−1^ MPA_bouts_ guideline level [6]–[8] is based on a compelling body of epidemiological and experimental evidence (summarised here [16]). However the data underpinning these recommendations is largely based on populations of white European origin, and it is acknowledged that further investigation is required to evaluate the relationship between physical activity and health status in non-Caucasian populations [16].

Our findings indicate that the threshold level of MPA_bouts_ in South Asians, equivalent to an MPA_bouts_ level of 150 min.week^−1^ in Europeans, is significantly higher for the glycaemia (299 min.week^−1^), insulin resistance (283 min.week^−1^) and lipid (364 min.week^−1^) factors. This suggests that – analogous to ethnicity-specific obesity cut-points – ethnicity-specific physical activity recommendations are potentially warranted. As guidelines require a single threshold value to be adopted, we also included a summary overall cardio-metabolic risk factor in our analyses which combined all the measured risk biomarkers. Physical activity explained more of the variance in this overall cardio-metabolic factor than the individual glycaemia, insulin resistance, lipid or blood pressure factors, which is consistent with the large body of evidence indicating that activity influences vascular and metabolic disease risk by multiple mechanisms [32]. Thus, we would argue that it is the threshold value based on this overall cardio-metabolic risk factor that should be used guide to any changes to physical activity recommendations to include an ethnicity-specific component. The equivalent MPA_bouts_ threshold in South Asians for the overall cardio-metabolic risk factor was 266 (228 to 313) min.week^−1^. This dropped to 197 (160 to 224) min.week^−1^ in sensitivity analyses where HbA1c was excluded from the model because of potential ethnic differences in HbA1c that are independent of glycaemia [29], [30]. Thus, in practical terms, a recommendation that South Asians undertake 200 to 250 min.week^−1^ – which equates to 40–50 minutes on five days of the week – of moderate-equivalent physical activity would seem to provide a clear, and easy to remember public health message.

A major strength of this study is the objective measurement of physical activity. This is essential to provide accurate quantification of the dose-response relationship between physical activity and cardio-metabolic risk. Previous reports have shown that questionnaire-based assessment of physical activity can overestimate activity levels by several fold compared to objective measures [25], [33] and the use of questionnaires to assess physical activity can substantially underestimate the true relationship between activity and cardio-metabolic disease risk [33], [34]. However, while use of a hip-mounted accelerometer is the most widely used method for the objective measure of physical activity, this approach does underestimate the level of activity associated with load-carrying, walking/running up a gradient or stairs, cycling or arm-based activities [25], and accelerometers cannot be worn during water-based activities, such as swimming. Thus, we may not have captured all relevant physical activity with our approach. However, we feel that this limitation is unlikely to have substantially influenced the overall findings, as all participants in this cohort completed a physical activity diary in parallel with the accelerometer measurements (data not shown) and only a small number of participants reported undertaking any swimming (n = 9) or cycling (n = 3) activity during the week of activity measurement. A further consideration is that we used the same cut-point to define moderate intensity physical activity, which was equivalent to an absolute intensity of ≥3 METS [24], in all participants. The South Asians in our cohort had ∼20% lower maximal oxygen uptake values than the Europeans (data reported in Ghouri et al [22]), and thus the physiological threshold for moderate intensity physical activity (in terms of relative exercise intensity) would have been lower in the South Asians than Europeans. Reducing the moderate intensity cut-point for the South Asians to take this into account would have increased the apparent amount of physical activity that the South Asians in this cohort undertook. However, doing this would also flatten the dose-response relationship between physical activity and cardio-metabolic risk biomarkers, leading to the threshold level of MPA_bouts_ in South Asians, equivalent to an MPA_bouts_ level of 150 min.week^−1^ in Europeans being higher than the 200-250 min.week^−1^ reported here.

In addition, the values for the South Asian obesity cut-points in the present study agreed well with those obtained in previous reports. The biomarkers included in the factor analysis models differed slightly between the studies of Razak [10], Gray [9] and the present report. For example, insulin was included in both the present and the Razak’s analysis, but not in the analysis of Gray and colleagues [9], and only Razak and colleagues [10] included free fatty acids in their model. Because of this, the biomarkers included within each summary factor differed slightly between the three studies: in our analyses the risk biomarkers clustered into four distinct factors – glycaemia, insulin resistance, lipids and blood pressure, in contrast to the earlier studies, where the risk biomarkers clustered into three separate factors representing glycaemia, lipids and blood pressure. However, the BMI and waist circumference cut-point values for the glycaemia and insulin resistance factors were very similar, so this difference in clustering does not substantially influence the overall message. Our cut-point estimates of 24.3 kg.m^−2^ for the glycaemia factor BMI cut-point and 23.3 kg.m^−2^ for the insulin resistance factor were similar to, and well within 95% confidence limits of, the glycaemia factor cut-points of 22.6 kg.m^−2^ reported by Gray et al [9] and the glucose factor cut-point of 21.0 kg.m^−2^ reported by Razak et al [10]. Similarly, our estimated cut-points for the lipid factor (23.3 kg.m^−2^) and blood pressure factor (28.3 kg.m^−2^) were similar to the equivalent cut points in the Gray (26.0 kg.m^−2^ and 28.4 kg.m^−2^, respectively) [9] and Razak (22.5 kg.m^−2^ and 28.8 kg.m^−2^, respectively) [10] analyses. Our estimated waist circumference cut-points for glycaemia/insulin resistance, lipid and blood pressure factors (90.4/87.0 cm, 85.3 cm and 100.7 cm, respectively) also agreed well (within 95% CI limits) with values reported by Gray and colleagues (83.8 cm, 91.4 cm and 99.3 cm, respectively) [9]. Thus, studies from three different populations of South Asians have all converged on similar obesity cut-points for this ethnic group, suggesting that these threshold estimates are likely to be robust, at least on the basis of cross-sectional data.

This is a relatively small proof-of-principle study aimed at demonstrating the potential need for ethnicity-specific physical activity guidelines. Our findings are limited to a relatively small cohort of non-diabetic middle-aged men, and the analyses were cross-sectional. Our relatively small sample size, together with the fact that the calculated equivalent MPA_bouts_ levels for South Asians were towards the upper end of the distribution of physical activity levels for our cohort (the maximum value for MPA_bouts_ was 383 min.week^−1^ in South Asians and 459 min.week^−1^ in Europeans), resulted in 95%CIs around our estimates that were relatively large. Thus, further work is needed to replicate our findings in larger cohorts, including both men and women, and ideally with prospective data using hard disease end-points as outcomes, before a definitive recommendation regarding ethnicity-specific physical activity guidelines can be made. Nevertheless, the present report describes a novel approach for determining equivalent physical activity levels in different ethnic groups and we hope that this work will help facilitate the larger definitive studies needed to influence guidelines and policy in practice.

In conclusion, the present findings provide evidence indicating that ethnicity-specific physical activity guidelines are conceptually warranted. While larger studies are need to confirm and replicate our findings, the present data suggest that for an equivalent cardio-metabolic risk profile to that observed in Europeans undertaking the currently recommended physical activity level of 150 min.week^−1^ of MPA_bouts_, South Asians need to undertake 200-250 min.week^−1^. This equates to South Asians undertaking an extra 10-20 minutes of physical activity per day beyond present physical activity guideline levels. Dissemination of this message in a positive manner could be used to promote increases in physical activity levels in South Asians worldwide, a group who, despite clearly having higher cardio-metabolic disease risks, appear to be (at least in the UK) currently less active than their white European counterparts [22], [35].

## References

[1] McGee DL (2005) Body mass index and mortality: a meta-analysis based on person-level data from twenty-six observational studies. Ann Epidemiol 15: 87–97. 15652713 10.1016/j.annepidem.2004.05.012

[2] Abdullah A, Peeters A, de Court, Stoelwinder J (2010) The magnitude of association between overweight and obesity and the risk of diabetes: a meta-analysis of prospective cohort studies. Diabetes Res Clin Pract 89: 309–319 S0168-8227(10)00194-4 [pii]; 10.1016/j.diabres.2010.04.012 [doi] 20493574

[3] Gill JM, Cooper AR (2008) Physical activity and prevention of type 2 diabetes mellitus. Sports Med 38: 807–824. 18803434 10.2165/00007256-200838100-00002

[4] Nocon M, Hiemann T, Muller-Riemenschneider F, Thalau F, Roll S, et al (2008) Association of physical activity with all-cause and cardiovascular mortality: a systematic review and meta-analysis. Eur J Cardiovasc Prev Rehabil 15: 239–246 10.1097/HJR.0b013e3282f55e09 [doi];00149831-200806000-00001 [pii] 18525377

[5] World Health Organisation (1998) Obesity: Preventing and Managing the Global Epidemic. Report on a WHO consultation on obesity. 11234459

[6] Department of Health (2011) Start Active, Stay Active: a report on physical activity for health from the four home countries' Chief Medical Officers. 1–59.

[7] U.S. Department of Health and Human Services (2008) 2008 Physical Activity Guidelines for Americans.

[8] World Health Organisation (2010) Global recommendations on physical activity for health. 1–58. 26180873

[9] Gray LJ, Yates T, Davies MJ, Brady E, Webb DR, et al (2011) Defining obesity cut-off points for migrant South asians. PLoS ONE 6: e26464 10.1371/journal.pone.0026464 [doi];PONE-D-11-06667 [pii] 22039493 PMC3198431

[10] Razak F, Anand SS, Shannon H, Vuksan V, Davis B, et al (2007) Defining obesity cut points in a multiethnic population. Circulation 115: 2111–2118. 17420343 10.1161/CIRCULATIONAHA.106.635011

[11] Misra A, Chowbey P, Makkar BM, Vikram NK, Wasir JS, et al (2009) Consensus statement for diagnosis of obesity, abdominal obesity and the metabolic syndrome for Asian Indians and recommendations for physical activity, medical and surgical management. J Assoc Physicians India 57: 163–170. 19582986

[12] Kumar S, Hanif W, Zaman MJ, Sattar N, Patel K, et al (2011) Lower thresholds for diagnosis and management of obesity in British South Asians. Int J Clin Pract 65: 375–385. 21401824 10.1111/j.1742-1241.2010.02548.x

[13] Cameron AJ, Sicree RA, Zimmet PZ, Alberti KG, Tonkin AM, et al (2010) Cut-points for waist circumference in Europids and South Asians. Obesity (Silver Spring ) 18: 2039–2046 oby2009455 [pii]; 10.1038/oby.2009.455 [doi] 20019679

[14] Alberti KG, Eckel RH, Grundy SM, Zimmet PZ, Cleeman JI, et al (2009) Harmonizing the metabolic syndrome: a joint interim statement of the International Diabetes Federation Task Force on Epidemiology and Prevention; National Heart, Lung, and Blood Institute; American Heart Association; World Heart Federation; International Atherosclerosis Society; and International Association for the Study of Obesity. Circulation 120: 1640–1645 CIRCULATIONAHA.109.192644 [pii]; 10.1161/CIRCULATIONAHA.109.192644 [doi] 19805654

[15] National Institute for Health and Care Excellence (2013) Assessing body mass index and waist circumference thresholds for intervening to prevent ill health and premature death among adults from black, Asian and other minority ethnic groups in the UK. guidance.nice.org.uk/ph46: 1–50.

[16] Warburton DE, Charlesworth S, Ivey A, Nettlefold L, Bredin SS (2010) A systematic review of the evidence for Canada's Physical Activity Guidelines for Adults. Int J Behav Nutr Phys Act 7: 39 1479-5868-7-39 [pii]; 10.1186/1479-5868-7-39 [doi] 20459783 PMC3583166

[17] Celis-Morales CA, Perez-Bravo F, Ibanes L, Sanzana R, Hormazabal E, et al (2011) Insulin resistance in Chileans of European and indigenous descent: evidence for an ethnicity x environment interaction. PLoS One 6: e24690 10.1371/journal.pone.0024690 [doi];PONE-D-11-02660 [pii] 21931814 PMC3169638

[18] Hall LML, Sattar N, Gill JMR (2008) Risk of metabolic and vascular disease in South Asians: potential mechanisms for increased insulin resistance. Future Lipidology 3: 411–424.

[19] Hall LM, Moran CN, Milne GR, Wilson J, MacFarlane NG, et al (2010) Fat oxidation, fitness and skeletal muscle expression of oxidative/lipid metabolism genes in South Asians: implications for insulin resistance? PLoS ONE 5: e14197 10.1371/journal.pone.0014197 [doi] 21152018 PMC2995737

[20] Davey GJG, Roberts JD, Patel S, Pierpoint T, Godsland IF, et al (2000) Effects of exercise on insulin resistance in South Asians and Europeans. Journal of Exercise Physiology 3: 6–11.

[21] Hardy CP, Eston RG (1985) Aerobic fitness of Anglo-Saxon and Indian students. Br J Sports Med 19: 217–218. 4092143 10.1136/bjsm.19.4.217 PMC1478397

[22] Ghouri N, Purves D, McConnachie A, Wilson J, Gill JM, et al (2013) Lower cardiorespiratory fitness contributes to increased insulin resistance and fasting glycaemia in middle-aged South Asian compared with European men living in the UK. Diabetologia 56: 2238–2249 10.1007/s00125-013-2969-y [doi] 23811809 PMC3764328

[23] Marfell-Jones M, Olds T, Stewart A, Carter L (2006) International standards for anthropometric assessment. PotchefstroomSouth Africa: ISAK. 133 p.

[24] Freedson PS, Melanson E, Sirard J (1998) Calibration of the Computer Science and Applications, Inc. accelerometer. Med Sci Sports Exerc 30: 777–781. 9588623 10.1097/00005768-199805000-00021

[25] Troiano RP, Berrigan D, Dodd KW, Masse LC, Tilert T, et al (2008) Physical activity in the United States measured by accelerometer. Med Sci Sports Exerc 40: 181–188 10.1249/mss.0b013e31815a51b3 [doi] 18091006

[26] El Assaad MA, Topouchian JA, Asmar RG (2003) Evaluation of two devices for self-measurement of blood pressure according to the international protocol: the Omron M5-I and the Omron 705IT. Blood Press Monit 8: 127–133 10.1097/01.mbp.0000087393.96145.b1 [doi] 12900590

[27] Herder C, Karakas M, Koenig W (2011) Biomarkers for the prediction of type 2 diabetes and cardiovascular disease. Clin Pharmacol Ther 90: 52–66 clpt201193 [pii]; 10.1038/clpt.2011.93 [doi] 21654741

[28] Sattar N (2012) Biomarkers for diabetes prediction, pathogenesis or pharmacotherapy guidance? Past, present and future possibilities. Diabet Med 29: 5–13 10.1111/j.1464-5491.2011.03480.x [doi] 21988593

[29] Mostafa SA, Davies MJ, Webb DR, Srinivasan BT, Gray LJ, et al (2012) Independent effect of ethnicity on glycemia in South Asians and white Europeans. Diabetes Care 35: 1746–1748 dc11-2079 [pii]; 10.2337/dc11-2079 [doi] 22699291 PMC3402276

[30] Herman WH, Cohen RM (2012) Racial and ethnic differences in the relationship between HbA1c and blood glucose: implications for the diagnosis of diabetes. J Clin Endocrinol Metab 97: 1067–1072 jc.2011-1894 [pii]; 10.1210/jc.2011-1894 [doi] 22238408 PMC3319188

[31] Wen CP, Wu X (2012) Stressing harms of physical inactivity to promote exercise. Lancet 380: 192–193 S0140-6736(12)60954-4 [pii]; 10.1016/S0140-6736(12)60954-4 [doi] 22818933

[32] Gill JM, Malkova D (2006) Physical activity, fitness and cardiovascular disease risk in adults: interactions with insulin resistance and obesity. Clin Sci (Lond) 110: 409–425. 16526946 10.1042/CS20050207

[33] Celis-Morales CA, Perez-Bravo F, Ibanez L, Salas C, Bailey ME, et al (2012) Objective vs. Self-Reported Physical Activity and Sedentary Time: Effects of Measurement Method on Relationships with Risk Biomarkers. PLoS ONE 7: e36345 10.1371/journal.pone.0036345 [doi];PONE-D-12-06438 [pii] 22590532 PMC3348936

[34] Atienza AA, Moser RP, Perna F, Dodd K, Ballard-Barbash R, et al (2011) Self-reported and objectively measured activity related to biomarkers using NHANES. Med Sci Sports Exerc 43: 815–821 10.1249/MSS.0b013e3181fdfc32 [doi] 20962693

[35] Fischbacher CM, Hunt S, Alexander L (2004) How physically active are South Asians in the United Kingdom? A literature review. J Public Health (Oxf) 26: 250–258 10.1093/pubmed/fdh158 [doi];26/3/250 [pii] 15454592

